# *Streptococcus gallolyticus* infection in colorectal cancer and association with biological and clinical factors

**DOI:** 10.1371/journal.pone.0174305

**Published:** 2017-03-29

**Authors:** Maria Andres-Franch, Antonio Galiana, Victoria Sanchez-Hellin, Enrique Ochoa, Eva Hernandez-Illan, Pilar Lopez-Garcia, Adela Castillejo, Maria Isabel Castillejo, Victor Manuel Barbera, Josefa Garcia-Dura, Francisco Javier Gomez-Romero, Gloria Royo, Jose Luis Soto

**Affiliations:** 1 Microbiology Dept., Hospital General Universitario de Elche, Elche, Spain; 2 Biopathology Dept., Hospital Provincial de Castellón, Castellón, Spain; 3 Research Lab, Hospital General Universitario de Alicante, Alicante, Spain; 4 Instituto de Investigación Sanitaria y Biómedica de Alicante (ISABIAL)–FISABIO, Alicante, Spain; 5 Molecular Genetics Unit, Hospital General Universitario de Elche, Elche, Spain; 6 Preventive Medicine Dept., Hospital General Universitario de Elche, Elche, Spain; Universidad de Chile, CHILE

## Abstract

There is an unambiguous association of *Streptococcus gallolyticus* infection with colorectal cancer, although there is limited information about epidemiology or interaction between molecular and environmental factors. We performed an original quantitative analysis of *S*. *gallolyticus* in unselected colorectal cancer patients (n = 190) and their association with clinical, pathological tumor molecular profiles (microsatellite instability, hypermethylator phenotype and chromosomal instability pathways), and other biological factors in colorectal tumor and normal tissues (cytomegalovirus and Epstein-Barr virus infection). We developed a new quantitative method to assess bacterial load. Analytical validation was reached with a very high sensitivity and specificity. Our results showed a 3.2% prevalence of *S*. *gallolyticus* infection in our unselected cohort of colorectal cancer cases (6/190). The average *S*. *gallolyticus* copy number was 7,018 (range 44–34,585). No previous reports relating to *S*. *gallolyticus* infection have been published for unselected cohorts of patients. Finally, and despite a low prevalence of *S*. *gallolyticus* in this study, we were able to define a specific association with tumor tissue (*p* = 0.03) and with coinfection with Epstein-Barr virus (*p* = 0.042; OR: 9.49; 95% IC: 1.1–82.9). The prevalence data provided will be very useful in the design of future studies, and will make it possible to estimate the sample size needed to assess precise objectives. In conclusion, our results show a low prevalence of *S*. *gallolyticus* infection in unselected colorectal cancer patients and an association of positive *S*. *gallolyticus* infection with tumor tissue and Epstein-Barr virus coinfection. Further studies will be needed to definitively assess the prevalence of *S*. *gallolyticus* in colorectal cancer and the associated clinicopathological and molecular profiles.

## Introduction

Cancer is a multifactorial group of diseases generated by a combination of different genetic and environmental factors. In the last decade we have experienced an exponential increase in new knowledge about the molecular basis of the disease because of the availability of new technological tools for massive molecular analysis. Nevertheless, there is ample room for improvement in the knowledge about the interaction between the molecular and environmental factors. Colorectal cancer (CRC) is the leading cause of cancer in westernized countries [1]. The main molecular features of CRC are well known, and a multistep process with a progressive accumulation of (epi)genetic alterations has been established as the route of carcinogenesis. Three major molecular pathways of carcinogenesis have been defined for tumor classification: microsatellite instability (MSI) group represented by 15% of CRC. This group includes defective DNA mismatch repair with microsatellite instability (MSI) and *POLE/POLD1* mutations, containing multiple frameshifted genes and *BRAFV600E* and is characterized by hypermutated tumors [2]; chromosomal instability (CIN) group represented by 85% of CRC. This is a non-hypermutated group with multiple somatic copy number alterations, and aneuploidy by recurrent missegregation of whole chromosomes during cell division [3,4], containing oncogenic activation of *KRAS* and *PIK3CA* and mutation and loss of heterozygosity of tumor suppressor genes such as *APC* and *TP53*; and CpG Island Methylator Phenotype (CIMP) CRCs in 20% that overlap greatly with MSI CRC and some non-hypermutated CRC [2].

The main environmental factors linked with CRC are diet and the closely related factor of the gut microbiota, including viruses. The gut microbiota is currently considered to be an organ, and the symbiotic interactions between the gut microbiota and the digestive tract, under the surveillance of the immune system, are essential for maintaining homeostasis. Any disruptive imbalance can alter this particular ecosystem and promote diseases such as inflammatory bowel diseases and cancer [5]. Many changes in the relative bacterial content of the gut have been described in CRC, suggesting a major role of dysbiosis in carcinogenesis. Among the dysbiotic bacterial species identified and suspected to play a role in colorectal carcinogenesis are *Streptococcus gallolyticus*, *Bacteroides fragilis*, *Enterococcus faecalis*, *Clostridium septicum*, *Fusobacterium* spp. and *Escherichia coli* [5]. Strikingly, however, *Streptococcus gallolyticus* subsp. *gallolyticus* (SG; previously known as *Streptococcus bovis* biotype I) is one of the very few opportunistic pathogens that has been clinically linked to malignant colonic diseases [6].

Regarding the role of viruses in CRC, a recent comprehensive screening for viruses using next-generation sequencing data from The Cancer Genome Atlas (TCGA) demonstrated the presence of viral sequences in CRC. Epstein–Barr virus (EBV), cytomegalovirus (CMV), and human papillomavirus type 18 (HPV-18) are considered potential causes of CRC, although their oncogenic role is yet to be established [7].

The results of a recent meta-analysis suggest an unambiguous association of SG infection with CRC. It has been proposed that colonization of the colonic mucosa by this bacterium could be a risk factor for CRC, although the nature of this association remains unknown [8]. There is a growing need to clarify whether it is cause or consequence and to provide information about the possible mechanisms involved. Available data are scarce and few reports have attempted a prevalence study of SG in unselected cases of CRC. In addition, there is limited information about the tumor molecular profile of SG-positive CRC cases that could reveal mechanistic clues for the potential role of SG in carcinogenesis, or alternatively, its oncomodulator effect in specific molecular subtypes of CRC.

The present study addresses the following two questions: (i) what is the prevalence of SG in tumor and normal mucosa from an unselected cohort of CRC patients, and (ii) what is the association, if any, of SG infection with clinical, pathological, molecular and other biological variables of the colorectal tumors.

## Materials and methods

### Design and analytical validation of a quantitative real-time PCR assay for *S*. *Gallolyticus* (SG) bacterial load

A collection of 76 isolated from bacteraemia from the Hospital General Universitario de Elche (Spain) was used in the present study to obtain SG-positive controls. The strains were originally identified as *Streptococcus bovis* with the commercial kit API 20 Strep (bioMérieux, Marcy l'Etoile, France). Strains were streaked onto chocolate agar plates and incubated at 37°C for 24 hours. One colony of each plate was selected and DNA was extracted using Chelex (Bio-Rad, Hercules, California, USA) according to the manufacturer’s instructions. Specific PCR primers for *SodA* gene were designed by the alignment of *SodA* gene sequences of *S*. *infantarius*, *S*. *lutetiensis* and SG giving an amplicon of 419-bp size. Primers: sodA_F: YGATRCAGAAACAATGACATTDCA; sodA_R: ATTGRTTYYTTACCYTCTGA. PCR conditions: initial denaturation step at 94°C for 2 min; followed by 30 cycles of denaturation at 94°C for 10 s, annealing at 50°C for 30 s, extension at 68°C for 30 s, and a final extension step at 68°C for 7 min. Amplification products were visualized in a 2% agarose gel. Sanger sequencing of the amplicons was performed to confirm SG strains (Secugen. Madrid, Spain). Sequence homologies were established by BLASTn (http://blast.st-va.ncbi.nlm.nih.gov/Blast.cgi). The SG-positive strains were selected as positive controls. Next, primers and TaqMan probes for qPCR to amplify the three subspecies of *S*. *gallolyticus*, specifically: *S*. *gallolyticus* subspp. *gallolyticus*, *S*. *gallolyticus* subspp. *pasteurianus* and *S*. *gallolyticus* subspp. *macedonicus* were designed according to the nucleotide sequences of *SodA* gene (Beacon Designer): Forward primer SG_F (5´-TGGCTCATTTGAYGAATT-3´), reverse primer SG_R (5´-GAGAGCACTTCAAGTTTG-3´) and probe SG (5´-FAM-TTCATTCACCACAAGCCA-BHQ1-3´) were used for detection. Real-time PCR reactions were prepared as follow: 12.5 μL of Universal PCR MasterMix (Applied Biosystems), 0.75 μL of 12.5 pmol/μL reverse and forward primers, 0.25 μL of probe 4 pmol/μL, 5 μL DNA and RNase free water up to 25 μL of the total reaction volume. PCR cycling conditions were 95°C for 10 min and 40 cycles of two steps of amplification (95°C for 15 s and 60°C for 1 min). SG qPCR detection assays were run in triplicate.

Standard curves and the limit of detection were obtained by plotting the threshold cycle value (Ct) obtained by a 10-fold serially diluted DNA against DNA copy number input. A standard curve was accepted when the slope was from –3.3 to –3.6. We used DNA from previously confirmed positive controls of *S*. *gallolyticus* and diluted from 10^6^−10 copies/μL. In addition, to assess the specificity of the quantitative PCR system, 20 different isolates of confirmed SG were analysed in triplicate. Reproducibility of results was assessed in independent validation experiments.

### Patients and specimens

A total of 380 samples derived from 190 patients (frozen tumor tissues and normal appearing colonic mucosa for each patient) from the Hospital Provincial Castellon Biobank (Spain) were included in this study. This is an unselected cohort of CRC patients who underwent surgery with curative intention.

In addition to the patients’ biological samples, clinical and pathological information were obtained from the Hospital Provincial Castellon Biobank. Written informed consent, for inclusion in the Biobank was obtained from every participating individual. The study complied with the Declaration of Helsinki (2013) and was approved by the Ethics Committee of the Elche University Hospital.

DNA was isolated from frozen colorectal normal and tumor tissues after mechanical homogenization (Tissue Lyser; Qiagen, Valencia, CA). DNA isolation was performed using the EZ1 DNA Tissue kit and the EZ1 BioRobot (Qiagen, Valencia, CA) according to the manufacturer’s instructions.

### Detection and quantification of *S*. *gallolyticus* in normal colonic mucosa and colorectal tumor tissues by qPCR

Quantitative assessment of SG in colorectal tissues was approached as described above. Fifty nanograms of DNA from each tissue sample at 1ng/μL were tested. In addition, positive and negative controls and a six-point standard curve (10–1,000,000 copies; Pearson’s correlation coefficient > 0.98) were analysed together with the samples for each run. Cycle threshold (Ct) values obtained by each sample were interpolated with the linear regression of the standard curve for the quantification. All the samples were analysed in triplicate. Positive results were considered when at least two of the replicates showed detectable bacterial load with more than 10 copies.

### Demographic, clinical and pathological variables

Demographic (gender and age), clinical and pathological variables (tumor location and stage) were collected as associated information from the Hospital Provincial Castellon Biobank. Tumors located in the caecum through the splenic flexure were grouped together as proximal colon cancers (P); tumors located in the descending, sigmoid colon, recto-sigmoid junction and rectum, were classified together as distal colon cancer (D). Stage at diagnosis was classified as I, II, III and IV [9].

### CRC molecular variables

To attempt a tumor molecular classification, the main molecular hallmarks of the different pathways were considered in the study. Therefore, CIMP phenotype and MSI status were tested to group the tumors in three mayor pathways as follow: (i) Chromosomal instability tumors were defined as microsatellite stable tumors and no hypermethylated phenotype; (ii) Microsatellite unstable tumors were those with MSI, independently of the methylation status, and (iii) tumors with hypermethylated phenotype were considered those with CIMP positive, and microsatellite stable results.

#### DNA tumor methylation status

The DNA tumor methylation status in CIMP was assessed by methylation-specific multiplex ligation-dependent probe amplification (MS-MLPA) using the SALSA MLPA ME042 CIMP probemix (MRC-Holland, Amsterdam, The Netherlands) according to the manufacturer’s protocol. This probemix contains 31 MS-MLPA probes which detect the methylation status of promoter regions of the following eight genes: *CACNA1G*, *CDKN2A*, *CRABP1*, *IGF2*, *MLH1*, *NEUROG1*, *RUNX3* and *SOCS1*. An altered methylation in these genes has been reported in the literature and has been used to test CIMP status [9]. The dichotomization threshold to distinguish methylated versus non-methylated samples was established at 20%. Tumors were considered as CIMP positive when at least five genes showed methylation over the established threshold in at least one of their probes [10].

#### Microsatellite instability analysis

Colorectal tumor DNAs were tested for MSI using multiplex-PCR for five mononucleotide quasimonomorphic markers (BAT25, BAT26, NR21, NR24 and NR27) and molecular analysis of fragments through capillary electrophoresis as previously described by Buhard et al [11]. Tumors were classified as MSI when at least two of the markers showed an altered peak pattern.

#### Qualitative analysis of EBV and CMV in normal and colorectal tumor tissues

Detection of EBV and CMV in normal colon and tumor tissues was approached by conventional singleplex PCR in a 45-cycle reaction to improve the sensitivity of the assay [12]. PCR primers and conditions are shown in S1 Table. All the DNAs were tested in triplicate, and positive and negative DNA controls were included for each run. Analyses of *TP53* and β*-globin* human genes were tested in parallel as controls for each DNA. A GeneAmp PCR System 9700 (Applied Biosystems) was used for PCR reactions, and electrophoresis to visualize amplification products in ethidium bromide-stained agarose gels. Positive results were considered when at least two of the three PCR replicates rendered a visible amplicon band with the expected size. To assess the specificity of the assay, 10 consecutive positive results for each virus were sequenced forward and reverse with a 3130 Genetic Analyser (Applied Biosystems), and the sequences’ results were aligned by BLASTn (http://blast.st-va.ncbi.nlm.nih.gov/Blast.cgi) for verification.

### Statistical analysis

The applied statistical tests were bilateral and significance was established at *p* < 0.05. The baseline characteristics of patients are presented as relative frequencies and were compared using χ^2^ tests or Fisher's exact test if necessary for categorical variables. The normality of the distribution of continuous variables was checked with the Shapiro–Wilk test, then they were described as means and standard deviation (SD) or medians and interquartile ranges (IR) and compared with the SG results using the Student’s *t* test or Mann–Whitney *U* test as appropriate. We estimated odds ratios (ORs) and 95% confidence intervals (CI) using multivariable adjusted logistic regression models. The models were controlled for potential confounders based on published factors and those variables with *p* values < 0.20 in the bivariate analysis.

The statistical analysis was performed using the statistical software R 3.3.0 (R Foundation for Statistical Computing, Vienna, Austria; http://www.r-project.org).

## Results

Analytical validation of the method for quantitating SG indicated sensitivity and specificity of 100% with a limit detection of 10 copies of bacterial genomic DNA (data not shown). All 20 SG isolates used as positive controls were identified and good reproducibility between experiments was achieved.

Overall, the median age of patients at diagnosis was 70 (range: 30–94) years and 95 (50%) were women. Thirty-eight cases of proximal colon cancer (20%) and 118 of distal colon cancer (62%) were observed. There were 42 (22%) cases at stage II and 48 (25%) at stage III. No data for tumor location or staging were available for 34 and 100 patients, respectively.

Six of the 190 patients included (3.2%) were positive for SG. All the positive cases were identified from tumor tissue samples, while none of the normal mucosa samples showed detectable SG DNA (*p* = 0.03). No other significant association was found for SG. The results of the bivariant analysis are shown in Table 1.

**Table 1:** Clinicopathological, molecular and biological features of included patients stratified by *Streptococcus gallolyticus* infection.

Total patients (n = 190)	n (%) of positive SG6 (3.2)	n (%) of negative SG184 (96.8)	*p-value
**Gender**			0.09
Male	5 (2.6)	90 (47.4)	
Female	1 (0.5)	94 (49.5)	
Age (mean ± SD)	67.6 (8.38)	70.1 (11.4)	0.43
**Type of tissue** (n = 380)			0.03
Normal	0 (0)	190 (50)	
Tumoral	6 (1.6)	184 (48.4)	
**Tumour location**			0.63
Proximal	2 (1.3)	36 (23.1)	
Distal	4 (2.6)	114 (73.1)	
**Tumour stage**			0.12
II	4 (4.4)	38 (42.2)	
III	1 (1.1)	47 (52.2)	
**CIMP status**			0.61
Positive	2 (1.1)	40 (21.4)	
Negative	4 (2.1)	141 (75.4)	
**Microsatellite phenotype**			0.42
MSI	0 (0)	18 (9.5)	
MSS	6 (3.2)	166 (87.4)	
**Molecular carcinogenetic pathways**			0.45
CIN	4 (2.1)	133 (71.1)	
CIMP	2 (1.1)	30 (16)	
MSI	0 (0)	18 (9.6)	
**CMV infection** (n = 380)			0.72
Positive	1 (0.3)	44 (11.8)	
Negative	5 (1.3)	324 (86.6)	
**EBV infection** (n = 380)			**0.01**
Positive	5 (1.3)	127 (34)	
Negative	1 (0.3)	241 (64.4)	

SG: *Streptococcus gallolyticus*; SD: Standard deviation; CIMP: CpG island methylator phenotype (CIMP)-positive tumor; MSI: Microsatellite instability; MSS: Microsatellite stability; CIN: Chromosomal instability; CMV: Cytomegalovirus; EBV: Epstein-Barr virus.

*P-values are from χ2 test (Fisher's exact test if necessary) or Mann-Whitney U test comparing *S*. *gallolyticus* results with categorical or continuous variables respectively.

The average SG copy number was 7,018 (range 44–34,585). In four of the six SG positive tumors, all three replicates were positive; while in the remaining two tumors only two of three replicates were considered positive. The results for these two samples were confirmed in an independent experiment.

The analysis of the molecular hallmarks of the tumors indicated that 9.5% of cases showed MSI (18/190) and 22.1% were CIMP-positive (CIMPpos) (42/190). Based on these results, a primary molecular pathway classification of tumors was established: 72.1% were classified as CIN (defined as CIMP negative (CIMPneg) and microsatellite-stable (MSS) tumors: 137/190); 9.5% as microsatellite unstable (MSI and any CIMP: 18/190); and 16.8% as CIMP (CIMPpos and MSS tumors: 32/190).

Tumors with MSI were associated with a proximal colon location (MSI-proximal (MSI-P): 10; MSI-distal (MSI-D): 5 *vs*. MSS-P: 28; MSS-D: 113; *p* < 0.001), earlier stages (MSI-Stage II: 10; MSI-Stage III: 2 *vs*. MSS-Stage II: 32; MSS-Stage III: 46; *p* = 0.002) and hypermethylation status (MSI-CIMPpos: 10; MSI-CIMPneg: 8 *vs*. MSS-CIMPpos: 32; MSS-CIMPneg: 108; *p* = 0.001).

EBV was detected in 52.4% of cases (99/189). In 63 of these cases, EBV was found only in tumor tissue; in 33 cases it was detected in both normal and tumor tissues, and in three cases it was found only in normal tissues (*p* < 0.001). CMV was detected in 22.8% of cases (43/189) and also was found significantly more often in tumor tissues than in normal tissue (43 tumor vs. 2 normal tissue, *p* < 0.001). Overall, a significant level of coinfection of CMV and EBV was found (*p* < 0.001). However, no association was observed when EBV–CMV coinfection was stratified by tissue type (normal vs. tumor).

In the logistic regression model, the presence of SG was associated with EBV infection (*p* = 0.042; OR: 9.49; 95% IC: 1.1–82.9) while the remaining variables were discarded in the adjustment process. Details of the logistic regression are shown in S2 Table. A description of the SG-positive cases identified in this study is shown in Table 2.

**Table 2:** Description of the *Streptococcus gallolyticus*-positive cases identified in the study.

Case	Gender	Age	EBV co-infection	CMV co-infection	CIMP status	Tumour location	Stage	Microsatellite phenotype	Mean Qty
1	Male	62	+	-	P	Distal	III	MSS	840
2	Male	63	-	-	P	Proximal	II	MSS	149
3	Male	83	+	+	N	Distal	II	MSS	1,390
4	Female	72	+	-	N	Distal	-	MSS	34,585
5	Male	63	+	-	N	Distal	II	MSS	5,102
6	Male	63	+	-	N	Proximal	II	MSS	44

EBV: Epstein-Barr virus; CMV: Cytomegalovirus; “P” indicates CpG island methylator phenotype (CIMP)-positive tumour; “N” indicates CpG island methylator phenotype (CIMP)-negative tumour; MSS: Microsatellite stability; Mean Qty: Mean of *S*. *gallolyticus* number of copies.

S3 Table shows the results for the analyzed variables for the whole series of cases.

## Discussion

The present study describes new insights into the nature of SG infection and its association with CRC. First, we developed a new quantitative method to assess bacterial load. Analytical validation was reached with a very high sensitivity and specificity. Second, we found a 3.2% prevalence of SG infection in our unselected cohort of CRC cases. No previous reports relating to SG infection have been published for unselected cohorts of patients. Third, to further characterize the nature of SG infection we performed an association study with clinical, pathological, molecular and other biological variables. Despite a low prevalence of SG in this study, we were able to define a specific association with tumor tissue and with coinfection with EBV.

The course of evolution of CRC depends on a combination of intrinsic and extrinsic factors. Therefore, the (epi)genetic background involving the main molecular factors that define different oncogenic pathways, together with lifestyle and microenvironmental (gut microbiota) factors and tumor treatment in combination with immune surveillance and immune responses, identify a complex combination of factors that defines the selective pressures that will determine the course of the disease [13].

To our knowledge, this is the first report in which SG in CRC patients is explored in relation to the molecular pathways of oncogenesis and concomitant viral infections. We hypothesized that this approach could be useful for providing information about the potential causal role of SG infection in tumorigenesis and the mechanisms involved.

Our results show a low prevalence (3.2%) of SG infection in patients with CRC compared with that reported in other studies [8]. Abdulamir et al. [14] reported a higher prevalence of SG in CRC patients: up to 49% in tumor tissue and 36% in the corresponding normal colonic mucosa. However, in that article, the authors focused on the study of CRC and normal tissues from a selected population with a history of SG bacteremia, which is a quite different scenario to the unselected series of CRC patients included in our study. Moreover, differences in the methodological aspects related to the detection of SG may contribute to the apparent discrepancy in the results.

The quantitative analysis of SG identified a broad range of bacterial loads (44–34,585 copies). For subsequent association analysis we dichotomized the results as SG-positive and SG-negative. It is very likely that a putative biological effect of SG on the tumor could be influenced by the bacterial load. We used 50 ng of DNA isolated from tumor tissues to establish the bacterial load, which represents a total of 8,000–10,000 tumor cells. Thus, the estimated ratio of copies of SG/tumor cell ranged from 5:1,000 to 4:1. It would be interesting to explore the pathological and clinical significance of the SG/tumor cell ratio in a larger series to obtain a more precise picture.

Despite the low prevalence of SG infection found in this unselected series of CRC patients, our results suggest that SG may play a role in CRC carcinogenesis and/or tumor progression because of the lack of detection of the bacterium in the normal colon mucosa. Similarly, our findings that EBV and CMV infections were preferentially found in tumor tissues may reflect their potential role in CRC oncogenesis. These results are consistent with those previously reported by Salyakina and Tsinoremas [7]. EBV is potentially causal in gastrointestinal cancer because it encodes oncoproteins and is able to transform human cells [15], while the oncogenic nature of CMV is still debatable [5].

MSI is a very well established marker used in clinical settings as diagnostic of Lynch syndrome, a good prognostic factor, and even as a predictor of treatment response [16]. In contrast, assessment of CIMP status is not conventional and is only used in a research context. The prognostic value of CIMP positivity is not known. Most studies show a worse prognosis for CIMPpos CRC, although adjuvant treatment seems to be more efficient in these tumors [17]. The methodology we used and the criteria we used to define CIMPpos tumors are restrictive enough to provide high specificity [18]. The frequencies of MSI and CIMP and the pathway distribution were concordant with those reported in the literature [2, 3]. Furthermore, the results obtained in this cohort support the well-established association of MSI tumors with a proximal colon location, earlier stages and hypermethylation status [19].

Despite the low number of cases positive for SG, we were able to identify its strong association with EBV coinfection and its presence specifically in tumor tissue, factors which have not been described previously. However, we still do not have enough information to establish whether SG infection is a cause (with an adjuvant or oncomodulator effect) or a consequence of the carcinogenic process. Further studies are needed to establish the association of SG with specific clinicopathological and molecular profiles of tumors to define its potential role in oncogenesis.

We acknowledge the limitations of this study. The limited sample size used in this work and the considerable amount of missing clinical and pathological data made it difficult to achieve results with high statistical power (potency of 72.5%). Consequently, we must be cautious with the interpretation of the results and consider them only as a proof of concept.

Nevertheless, it is important to note that the prevalence data provided by this study for SG infection in the tumors of an unselected cohort of CRC patients will be very useful in the design of future studies, and will make it possible to estimate the sample size needed to assess precise objectives.

## Conclusion

Our results show a low prevalence of SG infection in a cohort of unselected CRC patients and an association of positive SG infection with tumor tissue and EBV coinfection. Further studies will be needed to definitively assess the prevalence of SG in CRC and the associated clinicopathological and molecular profiles.

## Supporting information

S1 Table Primers and PCR conditions for Epstein–Barr virus and cytomegalovirus detection. (DOCX)

S2 Table Logistic regression analysis. (DOCX)

S3 Table Study database. Individual results.
(DOCX)
