# Supplementary material for: Streptococcus gallolyticus infection in colorectal cancer and association with biological and clinical factors
Source: PLoS One. 2017 Mar 29;12(3):e0174305. doi: 10.1371/journal.pone.0174305 (PMC5371321; doi:10.1371/journal.pone.0174305)
Supplement: S1 Table — (DOCX) [file pone.0174305.s001.docx]

**S1 Table**. Primers and PCR conditions for Epstein-Barr virus and cytomegalovirus detection.

| Epstein-Barr virus | Forward primer: AACATTGGCAGCAGGTAAGC  Reverse primer: ACTTACCAAGTGTCCATAGGAGC |
| --- | --- |
|  | PCR conditions: 95ºC-8min; [95ºC-30 s; 55ºC-30 s; 72ºC-30 s]x5; [95ºC-30 s; 60ºC-30 s; 72ºC-30 s]x40; 72ºC-10 min |
|  | Amplicon size: 186 bp |
| Cytomegalovirus | Forward primer: GTACACGCACGCTGGTTACC  Reverse primer: GTAGAAAGCCTCGACATCGC |
|  | PCR conditions: 95ºC-8min; [95ºC-30 s; 55ºC-30 s; 72ºC-30 s]x5; [95ºC-30 s; 60ºC-30 s; 72ºC-30 s]x40; 72ºC-10 min |
|  | Amplicon size: 257 bp |
