# Supplementary material for: Streptococcus gallolyticus infection in colorectal cancer and association with biological and clinical factors
Source: PLoS One. 2017 Mar 29;12(3):e0174305. doi: 10.1371/journal.pone.0174305 (PMC5371321; doi:10.1371/journal.pone.0174305)
Supplement: S2 Table — (DOCX) [file pone.0174305.s002.docx]

**S2 Table**. Logistic Regression Analysis.

While model 1 (GLM.1) was statistically significant from both the model and the variable EBV, model 2 (GLM.2) was significant in the ANOVA test but not through stepwise regression, and models 3 (GLM.3) and 4 (GLM.4) were not better than model 1. So, we concluded that model 1 was the final model.

| Model 1 | **> GLM.1 <- glm(SG ~ EBV, family=binomial(logit), data=Prueba) summary(GLM.1)**  Coefficients:                     Estimate Std. Error z value   Pr(>\|z\|)  (Intercept)   -5.485      1.002      -5.473   4.41e-08 ***  **EBV[T.P]**       2.250      1.101     2.044    **0.042 ***  ---  Signif. codes:  0 '***' 0.001 '**' 0.01 '*' 0.05 '.' 0.1 ' ' 1 |
| --- | --- |
| Model 2 | **> GLM.2 <- glm(SG ~ EBV + Tissue, family=binomial(logit), data=Prueba)** **summary(GLM.2)**  Coefficients:                             Estimate Std. Error   z value   Pr(>\|z\|)  (Intercept)           -22.074   2077.394    -0.011    0.992  **EBV[T.P]                1.598      1.105      1.446     0.148**  **Tissue[T.Tumor]   17.574   2077.394   0.008    0.993**    **> anova(GLM.1, GLM.2, test="Chisq")** **Analysis of Deviance Table**  Model 1: SG ~ EBV  Model 2: SG ~ EBV + Tissue          Resid. Df Resid. Dev Df Deviance Pr(>Chi)  1       372     55.516  2       371     50.295        1   5.2208 **0.02232 *** |
| Model 3 | **> GLM.3 <- glm(SG ~ EBV + Tissue + Gender, family=binomial(logit), data=Prueba)**  **summary(GLM.3)**  Coefficients:                            Estimate Std.Error     z value Pr(>\|z\|)  (Intercept)           -23.026   2028.336  -0.011    0.991  EBV[T.P]               1.478   1.111          1.330    0.184  Tissue[T.Tumor]   17.545   2028.335   0.009    0.993  Gender[T.Male]     1.575      1.111   1.418    0.156    **> anova(GLM.2, GLM.3, test="Chisq")** **Analysis of Deviance Table**  Model 1: SG ~ EBV + Tissue  Model 2: SG ~ EBV + Tissue + Gender          Resid. Df Resid. Dev Df Deviance Pr(>Chi)  1       371     50.295  2       370     47.644          1   2.6508   **0.1035** |
| Model 4 | **> GLM.4 <- glm(SG ~ EBV + Tissue + Stage, family=binomial(logit), data=Prueba)**  **summary(GLM.4)**  Coefficients:                            Estimate    Std. Error   z value Pr(>\|z\|)  (Intercept)          -21.214     2936.336  -0.007   0.9942  **EBV[T.P]             1.912        1.161        1.648   0.0994 .**  **Tissue[T.Tumor]   17.889   2936.336   0.006   0.9951**  **Stage[T.III]             -1.870      1.161      -1.610   0.1073**  **> anova(GLM.2, GLM.4, test="Chisq")** **Analysis of Deviance Table**  Model 1: SG ~ EBV + Tissue  Model 2: SG ~ EBV + Tissue + Stage          Resid. Df Resid. Dev Df Deviance Pr(>Chi)  1       175     35.770  2       174     32.466         1    3.304          **0.06911** |
