# Supplementary material for: Streptococcus gallolyticus infection in colorectal cancer and association with biological and clinical factors
Source: PLoS One. 2017 Mar 29;12(3):e0174305. doi: 10.1371/journal.pone.0174305 (PMC5371321; doi:10.1371/journal.pone.0174305)
Supplement: S3 Table — Individual results. (DOCX) [file pone.0174305.s003.docx]

**S3 Table**. Study database. Individual results.

| **Case** | | **Tissue** | **Gender** | **Age** | **Location** | **Stage** | **CIMP*** | **MSI** | **Pathway** | **CMV** | **EBV** | **SG** | **SG load** |
| --- | --- | --- | --- | --- | --- | --- | --- | --- | --- | --- | --- | --- | --- |
| **1** | | Tumor | Male | 77 | Proximal | III | N | MSS | CIN | P | P | N |  |
|  | | Normal |  |  |  |  |  |  |  | N | N | N |  |
| **2** | | Tumor | Male | 60 | Distal | Unknown | P | MSS | CIMP | N | P | N |  |
|  | | Normal |  |  |  |  |  |  |  | N | P | N |  |
| **3** | | Tumor | Female | 87 | Distal | III | N | MSS | CIN | P | N | N |  |
|  | | Normal |  |  |  |  |  |  |  | N | N | N |  |
| **4** | | Tumor | Male | 70 | Proximal | Unknown | N | MSS | CIN | P | P | N |  |
|  | | Normal |  |  |  |  |  |  |  | N | N | N |  |
| **5** | | Tumor | Female | 81 | Proximal | II | P | MSI | MSI | P | N | N |  |
|  | | Normal |  |  |  |  |  |  |  | N | N | N |  |
| **6** | | Tumor | Male | 76 | Distal | Unknown | P | MSS | CIMP | N | P | N |  |
|  | | Normal |  |  |  |  |  |  |  | N | P | N |  |
| **7** | | Tumor | Male | 62 | Distal | III | P | MSS | CIMP | N | P | P | 840.00 |
|  | | Normal |  |  |  |  |  |  |  | N | P | N |  |
| **8** | | Tumor | Male | 75 | Distal | Unknown | N | MSS | CIN | N | P | N |  |
|  | | Normal |  |  |  |  |  |  |  | N | N | N |  |
| **9** | | Tumor | Female | 49 | Unknown | Unknown | N | MSS | CIN | N | N | N |  |
|  | | Normal |  |  |  |  |  |  |  | N | P | N |  |
| **10** | | Tumor | Female | 74 | Unknown | III | N | MSS | CIN | N | N | N |  |
|  | | Normal |  |  |  |  |  |  |  | N | P | N |  |
| **11** | | Tumor | Female | 69 | Distal | III | N | MSS | CIN | N | N | N |  |
|  | | Normal |  |  |  |  |  |  |  | N | N | N |  |
| **12** | | Tumor | Male | 81 | Distal | III | P | MSS | CIMP | N | N | N |  |
|  | | Normal |  |  |  |  |  |  |  | N | N | N |  |
| **13** | | Tumor | Female | 77 | Proximal | III | P | MSS | CIMP | N | P | N |  |
|  | | Normal |  |  |  |  |  |  |  | N | P | N |  |
| **14** | | Tumor | Female | 75 | Unknown | III | P | MSS | CIMP | N | N | N |  |
|  | | Normal |  |  |  |  |  |  |  | N | N | N |  |
| **15** | | Tumor | Male | 52 | Distal | Unknown | N | MSS | CIN | N | N | N |  |
|  | | Normal |  |  |  |  |  |  |  | N | N | N |  |
| **16** | | Tumor | Male | 80 | Unknown | III | N | MSS | CIN | N | N | N |  |
|  | | Normal |  |  |  |  |  |  |  | N | N | N |  |
| **17** | | Tumor | Female | 48 | Unknown | II | P | MSS | CIMP | N | P | N |  |
|  | | Normal |  |  |  |  |  |  |  | N | N | N |  |
| **18** | | Tumor | Male | 69 | Distal | II | N | MSS | CIN | N | N | N |  |
|  | | Normal |  |  |  |  |  |  |  | N | N | N |  |
| **19** | | Tumor | Female | 67 | Unknown | II | N | MSI | MSI | N | N | N |  |
|  | | Normal |  |  |  |  |  |  |  | N | N | N |  |
| **20** | | Tumor | Female | 72 | Proximal | Unknown | P | MSS | CIMP | P | P | N |  |
|  | | Normal |  |  |  |  |  |  |  | N | N | N |  |
| **21** | | Tumor | Male | 74 | Distal | Unknown | N | MSS | CIN | N | P | N |  |
|  | | Normal |  |  |  |  |  |  |  | N | P | N |  |
| **22** | | Tumor | Female | 83 | Distal | II | N | MSS | CIN | P | P | N |  |
|  | | Normal |  |  |  |  |  |  |  | N | N | N |  |
| **23** | | Tumor | Male | 60 | Distal | III | P | MSS | CIMP | N | N | N |  |
|  | | Normal |  |  |  |  |  |  |  | N | N | N |  |
| **24** | | Tumor | Male | 94 | Distal | Unknown | P | MSS | CIMP | P | N | N |  |
|  | | Normal |  |  |  |  |  |  |  | N | N | N |  |
| **25** | | Tumor | Male | 76 | Distal | Unknown | N | MSS | CIN | P | N | N |  |
|  | | Normal |  |  |  |  |  |  |  | N | N | N |  |
| **26** | | Tumor | Male | 81 | Distal | Unknown | P | MSS | CIMP | N | N | N |  |
|  | | Normal |  |  |  |  |  |  |  | na | na | N |  |
| **27** | | Tumor | Female | 61 | Proximal | II | N | MSS | CIN | N | P | N |  |
|  | | Normal |  |  |  |  |  |  |  | N | N | N |  |
| **28** | | Tumor | Male | 70 | Distal | II | P | MSS | CIMP | N | N | N |  |
|  | | Normal |  |  |  |  |  |  |  | N | N | N |  |
| **29** | | Tumor | Female | 89 | Proximal | III | N | MSS | CIN | N | N | N |  |
|  | | Normal |  |  |  |  |  |  |  | N | N | N |  |
| **30** | | Tumor | Male | 58 | Proximal | Unknown | P | MSS | CIMP | N | P | N |  |
|  | | Normal |  |  |  |  |  |  |  | N | N | N |  |
| **31** | | Tumor | Female | 74 | Unknown | II | N | MSS | CIN | N | N | N |  |
|  | | Normal |  |  |  |  |  |  |  | N | N | N |  |
| **32** | | Tumor | Male | 63 | Proximal | II | P | MSS | CIMP | N | N | P | 149.00 |
|  | | Normal |  |  |  |  |  |  |  | N | N | N |  |
| **33** | | Tumor | Male | 30 | Unknown | III | N | MSS | CIN | N | N | N |  |
|  | | Normal |  |  |  |  |  |  |  | N | N | N |  |
| **34** | | Tumor | Male | 75 | Distal | Unknown | N | MSS | CIN | N | N | N |  |
|  | | Normal |  |  |  |  |  |  |  | N | P | N |  |
| **35** | | Tumor | Male | 68 | Distal | Unknown | N | MSS | CIN | N | P | N |  |
|  | | Normal |  |  |  |  |  |  |  | N | P | N |  |
| **36** | | Tumor | Male | 76 | Distal | II | P | MSS | CIMP | P | N | N |  |
|  | | Normal |  |  |  |  |  |  |  | N | N | N |  |
| **37** | | Tumor | Male | 65 | Distal | II | N | MSS | CIN | N | N | N |  |
|  | | Normal |  |  |  |  |  |  |  | N | N | N |  |
| **38** | | Tumor | Male | 79 | Distal | II | N | MSS | CIN | N | N | N |  |
|  | | Normal |  |  |  |  |  |  |  | N | N | N |  |
| **39** | | Tumor | Male | 73 | Distal | Unknown | N | MSS | CIN | N | N | N |  |
|  | | Normal |  |  |  |  |  |  |  | N | N | N |  |
| **40** | | Tumor | Female | 59 | Distal | Unknown | N | MSS | CIN | P | P | N |  |
|  | | Normal |  |  |  |  |  |  |  | P | P | N |  |
| **41** | | Tumor | Male | 67 | Distal | II | N | MSS | CIN | N | N | N |  |
|  | | Normal |  |  |  |  |  |  |  | N | N | N |  |
| **42** | | Tumor | Male | 56 | Distal | Unknown | N | MSS | CIN | N | P | N |  |
|  | | Normal |  |  |  |  |  |  |  | N | N | N |  |
| **43** | | Tumor | Female | 61 | Distal | III | N | MSS | CIN | P | P | N |  |
|  | | Normal |  |  |  |  |  |  |  | N | N | N |  |
| **44** | | Tumor | Male | 66 | Distal | II | Unknown | MSS | na | na | na | N |  |
|  | | Normal |  |  |  |  |  |  |  | na | na | N |  |
| **45** | | Tumor | Male | 62 | Distal | Unknown | P | MSS | CIMP | N | N | N |  |
|  | | Normal |  |  |  |  |  |  |  | N | P | N |  |
| **46** | | Tumor | Female | 61 | Distal | II | N | MSS | CIN | P | N | N |  |
|  | | Normal |  |  |  |  |  |  |  | N | P | N |  |
| **47** | | Tumor | Female | 78 | Distal | Unknown | N | MSS | CIN | N | N | N |  |
|  | | Normal |  |  |  |  |  |  |  | N | N | N |  |
| **48** | | Tumor | Male | 67 | Distal | Unknown | Unknown | MSS | na | na | na | N |  |
|  | | Normal |  |  |  |  |  |  |  | na | na | N |  |
| **49** | | Tumor | Female | 72 | Proximal | II | N | MSS | CIN | N | N | N |  |
|  | | Normal |  |  |  |  |  |  |  | N | N | N |  |
| **50** | | Tumor | Male | 49 | Distal | III | P | MSS | CIMP | N | P | N |  |
|  | | Normal |  |  |  |  |  |  |  | N | N | N |  |
| **51** | | Tumor | Female | 71 | Proximal | III | N | MSS | CIN | P | P | N |  |
|  | | Normal |  |  |  |  |  |  |  | N | N | N |  |
| **52** | | Tumor | Female | 49 | Proximal | II | N | MSS | CIN | N | N | N |  |
|  | | Normal |  |  |  |  |  |  |  | N | N | N |  |
| **53** | | Tumor | Female | 78 | Proximal | III | N | MSS | CIN | P | N | N |  |
|  | | Normal |  |  |  |  |  |  |  | N | N | N |  |
| **54** | | Tumor | Female | 72 | Proximal | Unknown | N | MSS | CIN | N | N | N |  |
|  | | Normal |  |  |  |  |  |  |  | N | N | N |  |
| **55** | | Tumor | Male | 47 | Distal | III | N | MSS | CIN | N | N | N |  |
|  | | Normal |  |  |  |  |  |  |  | N | N | N |  |
| **56** | | Tumor | Female | 61 | Distal | Unknown | N | MSS | CIN | N | N | N |  |
|  | | Normal |  |  |  |  |  |  |  | N | N | N |  |
| **57** | | Tumor | Male | 64 | Unknown | Unknown | N | MSI | MSI | N | N | N |  |
|  | | Normal |  |  |  |  |  |  |  | N | N | N |  |
| **58** | | Tumor | Female | 85 | Distal | II | N | MSS | CIN | N | N | N |  |
|  | | Normal |  |  |  |  |  |  |  | N | N | N |  |
| **59** | | Tumor | Male | 63 | Distal | Unknown | P | MSS | CIMP | P | P | N |  |
|  | | Normal |  |  |  |  |  |  |  | N | N | N |  |
| **60** | | Tumor | Male | 63 | Proximal | II | N | MSS | CIN | N | P | P | 44.00 |
|  | | Normal |  |  |  |  |  |  |  | N | N | N |  |
| **61** | | Tumor | Male | 68 | Distal | II | N | MSS | CIN | P | N | N |  |
|  | | Normal |  |  |  |  |  |  |  | N | N | N |  |
| **62** | | Tumor | Male | 70 | Distal | Unknown | N | MSS | CIN | N | N | N |  |
|  | | Normal |  |  |  |  |  |  |  | N | N | N |  |
| **63** | | Tumor | Male | 83 | Distal | Unknown | N | MSS | CIN | P | N | N |  |
|  | | Normal |  |  |  |  |  |  |  | N | N | N |  |
| **64** | | Tumor | Male | 70 | Distal | III | P | MSS | CIMP | N | P | N |  |
|  | | Normal |  |  |  |  |  |  |  | N | N | N |  |
| **65** | | Tumor | Female | 66 | Distal | II | N | MSS | CIN | P | N | N |  |
|  | | Normal |  |  |  |  |  |  |  | N | N | N |  |
| **66** | | Tumor | Female | 89 | Proximal | II | P | MSI | MSI | N | P | N |  |
|  | | Normal |  |  |  |  |  |  |  | N | N | N |  |
| **67** | | Tumor | Male | 83 | Distal | Unknown | N | MSS | CIN | P | N | N |  |
|  | | Normal |  |  |  |  |  |  |  | N | N | N |  |
| **68** | | Tumor | Female | 69 | Proximal | III | N | MSS | CIN | N | N | N |  |
|  | | Normal |  |  |  |  |  |  |  | N | N | N |  |
| **69** | | Tumor | Female | 62 | Distal | Unknown | N | MSS | CIN | N | P | N |  |
|  | | Normal |  |  |  |  |  |  |  | N | N | N |  |
| **70** | | Tumor | Female | 73 | Distal | III | P | MSS | CIMP | N | N | N |  |
|  | | Normal |  |  |  |  |  |  |  | N | N | N |  |
| **71** | | Tumor | Male | 65 | Distal | III | N | MSS | CIN | P | N | N |  |
|  | | Normal |  |  |  |  |  |  |  | N | N | N |  |
| **72** | | Tumor | Male | 79 | Distal | III | P | MSI | MSI | N | P | N |  |
|  | | Normal |  |  |  |  |  |  |  | N | N | N |  |
| **73** | | Tumor | Male | 75 | Proximal | Unknown | N | MSS | CIN | N | P | N |  |
|  | | Normal |  |  |  |  |  |  |  | P | N | N |  |
| **74** | | Tumor | Female | 73 | Distal | III | N | MSS | CIN | N | P | N |  |
|  | | Normal |  |  |  |  |  |  |  | N | N | N |  |
| **75** | | Tumor | Male | 83 | Distal | II | N | MSS | CIN | P | P | P | 1,390.00 |
|  | | Normal |  |  |  |  |  |  |  | N | P | N |  |
| **76** | | Tumor | Female | 77 | Distal | II | N | MSI | MSI | N | N | N |  |
|  | | Normal |  |  |  |  |  |  |  | N | N | N |  |
| **77** | | Tumor | Male | 80 | Proximal | Unknown | N | MSS | CIN | N | P | N |  |
|  | | Normal |  |  |  |  |  |  |  | N | N | N |  |
| **78** | | Tumor | Female | 74 | Distal | Unknown | P | MSS | CIMP | N | N | N |  |
|  | | Normal |  |  |  |  |  |  |  | N | N | N |  |
| **79** | | Tumor | Female | 71 | Distal | Unknown | N | MSS | CIN | P | P | N |  |
|  | | Normal |  |  |  |  |  |  |  | N | P | N |  |
| **80** | | Tumor | Female | 75 | Distal | Unknown | N | MSS | CIN | N | N | N |  |
|  | | Normal |  |  |  |  |  |  |  | N | N | N |  |
| **81** | | Tumor | Male | 86 | Distal | III | N | MSS | CIN | N | P | N |  |
|  | | Normal |  |  |  |  |  |  |  | N | N | N |  |
| **82** | | Tumor | Male | 69 | Unknown | II | N | MSS | CIN | N | P | N |  |
|  | | Normal |  |  |  |  |  |  |  | N | N | N |  |
| **83** | | Tumor | Male | 72 | Distal | III | N | MSS | CIN | P | P | N |  |
|  | | Normal |  |  |  |  |  |  |  | N | N | N |  |
| **84** | | Tumor | Female | 71 | Proximal | Unknown | N | MSS | CIN | N | N | N |  |
|  | | Normal |  |  |  |  |  |  |  | N | N | N |  |
| **85** | | Tumor | Female | 93 | Distal | Unknown | N | MSS | CIN | N | N | N |  |
|  | | Normal |  |  |  |  |  |  |  | N | P | N |  |
| **86** | | Tumor | Male | 84 | Distal | II | N | MSI | MSI | N | P | N |  |
|  | | Normal |  |  |  |  |  |  |  | N | N | N |  |
| **87** | | Tumor | Female | 87 | Distal | Unknown | N | MSS | CIN | N | N | N |  |
|  | | Normal |  |  |  |  |  |  |  | N | N | N |  |
| **88** | | Tumor | Male | 82 | Distal | Unknown | N | MSS | CIN | P | P | N |  |
|  | | Normal |  |  |  |  |  |  |  | N | N | N |  |
| **89** | | Tumor | Male | 73 | Distal | III | N | MSS | CIN | P | P | N |  |
|  | | Normal |  |  |  |  |  |  |  | N | N | N |  |
| **90** | | Tumor | Male | 63 | Distal | III | N | MSS | CIN | N | P | N |  |
|  | | Normal |  |  |  |  |  |  |  | N | P | N |  |
| **91** | | Tumor | Male | 69 | Proximal | Unknown | N | MSS | CIN | N | P | N |  |
|  | | Normal |  |  |  |  |  |  |  | N | P | N |  |
| **92** | | Tumor | Female | 67 | Distal | Unknown | N | MSS | CIN | N | N | N |  |
|  | | Normal |  |  |  |  |  |  |  | N | N | N |  |
| **93** | | Tumor | Male | 75 | Distal | III | P | MSS | CIMP | P | P | N |  |
|  | | Normal |  |  |  |  |  |  |  | N | P | N |  |
| **94** | | Tumor | Male | 67 | Distal | II | N | MSS | CIN | N | N | N |  |
|  | | Normal |  |  |  |  |  |  |  | N | N | N |  |
| **95** | | Tumor | Female | 62 | Distal | III | P | MSS | CIMP | P | N | N |  |
|  | | Normal |  |  |  |  |  |  |  | N | N | N |  |
| **96** | | Tumor | Female | 56 | Proximal | III | N | MSS | CIN | N | N | N |  |
|  | | Normal |  |  |  |  |  |  |  | N | N | N |  |
| **97** | | Tumor | Female | 65 | Distal | III | N | MSS | CIN | N | P | N |  |
|  | | Normal |  |  |  |  |  |  |  | N | P | N |  |
| **98** | | Tumor | Female | 71 | Distal | III | N | MSS | CIN | N | P | N |  |
|  | | Normal |  |  |  |  |  |  |  | N | N | N |  |
| **99** | | Tumor | Female | 57 | Distal | Unknown | N | MSS | CIN | P | P | N |  |
|  | | Normal |  |  |  |  |  |  |  | N | N | N |  |
| **100** | | Tumor | Female | 77 | Proximal | III | N | MSI | MSI | N | P | N |  |
|  | | Normal |  |  |  |  |  |  |  | N | N | N |  |
| **101** | | Tumor | Female | 77 | Proximal | III | P | MSS | CIMP | N | N | N |  |
|  | | Normal |  |  |  |  |  |  |  | N | N | N |  |
| **102** | | Tumor | Male | 81 | Distal | Unknown | P | MSS | CIMP | N | P | N |  |
|  | | Normal |  |  |  |  |  |  |  | N | N | N |  |
| **103** | | Tumor | Female | 88 | Distal | Unknown | N | MSS | CIN | P | P | N |  |
|  | | Normal |  |  |  |  |  |  |  | N | P | N |  |
| **104** | | Tumor | Female | 66 | Proximal | II | N | MSI | MSI | N | N | N |  |
|  | | Normal |  |  |  |  |  |  |  | N | N | N |  |
| **105** | | Tumor | Female | 84 | Distal | III | N | MSS | CIN | N | P | N |  |
|  | | Normal |  |  |  |  |  |  |  | N | P | N |  |
| **106** | | Tumor | Female | 76 | Proximal | II | P | MSI | MSI | N | P | N |  |
|  | | Normal |  |  |  |  |  |  |  | N | N | N |  |
| **107** | | Tumor | Male | 70 | Distal | Unknown | N | MSS | CIN | N | P | N |  |
|  | | Normal |  |  |  |  |  |  |  | N | N | N |  |
| **108** | | Tumor | Female | 66 | Distal | III | N | MSS | CIN | N | N | N |  |
|  | | Normal |  |  |  |  |  |  |  | N | N | N |  |
| **109** | | Tumor | Male | 72 | Proximal | III | P | MSS | CIMP | N | N | N |  |
|  | | Normal |  |  |  |  |  |  |  | N | P | N |  |
| **110** | | Tumor | Female | 83 | Distal | II | N | MSS | CIN | N | P | N |  |
|  | | Normal |  |  |  |  |  |  |  | N | N | N |  |
| **111** | | Tumor | Male | 82 | Proximal | Unknown | N | MSS | CIN | N | N | N |  |
|  | | Normal |  |  |  |  |  |  |  | N | N | N |  |
| **112** | | Tumor | Male | 65 | Unknown | II | N | MSS | CIN | N | N | N |  |
|  | | Normal |  |  |  |  |  |  |  | N | N | N |  |
| **113** | | Tumor | Male | 84 | Proximal | III | N | MSS | CIN | N | N | N |  |
|  | | Normal |  |  |  |  |  |  |  | N | N | N |  |
| **114** | | Tumor | Male | 69 | Proximal | Unknown | N | MSS | CIN | N | N | N |  |
|  | | Normal |  |  |  |  |  |  |  | N | N | N |  |
| **115** | | Tumor | Male | 70 | Distal | Unknown | N | MSS | CIN | N | N | N |  |
|  | | Normal |  |  |  |  |  |  |  | N | N | N |  |
| **116** | | Tumor | Male | 69 | Proximal | Unknown | N | MSS | CIN | N | P | N |  |
|  | | Normal |  |  |  |  |  |  |  | N | N | N |  |
| **117** | | Tumor | Female | 79 | Proximal | Unknown | P | MSI | MSI | P | N | N |  |
|  | | Normal |  |  |  |  |  |  |  | N | N | N |  |
| **118** | | Tumor | Female | 66 | Proximal | Unknown | N | MSS | CIN | P | P | N |  |
|  | | Normal |  |  |  |  |  |  |  | N | N | N |  |
| **119** | | Tumor | Female | 54 | Distal | Unknown | N | MSS | CIN | N | N | N |  |
|  | | Normal |  |  |  |  |  |  |  | N | P | N |  |
| **120** | | Tumor | Male | 77 | Distal | III | N | MSS | CIN | N | N | N |  |
|  | | Normal |  |  |  |  |  |  |  | N | P | N |  |
| **121** | | Tumor | Male | 72 | Distal | Unknown | N | MSS | CIN | N | P | N |  |
|  | | Normal |  |  |  |  |  |  |  | N | N | N |  |
| **122** | | Tumor | Female | 74 | Distal | III | N | MSS | CIN | N | N | N |  |
|  | | Normal |  |  |  |  |  |  |  | N | P | N |  |
| **123** | | Tumor | Female | 79 | Proximal | Unknown | N | MSS | CIN | N | P | N |  |
|  | | Normal |  |  |  |  |  |  |  | N | N | N |  |
| **124** | | Tumor | Female | 74 | Distal | II | N | MSS | CIN | N | N | N |  |
|  | | Normal |  |  |  |  |  |  |  | N | N | N |  |
| **125** | | Tumor | Male | 53 | Distal | Unknown | N | MSS | CIN | N | N | N |  |
|  | | Normal |  |  |  |  |  |  |  | N | N | N |  |
| **126** | | Tumor | Male | 55 | Distal | II | N | MSS | CIN | N | P | N |  |
|  | | Normal |  |  |  |  |  |  |  | N | N | N |  |
| **127** | | Tumor | Female | 64 | Distal | Unknown | N | MSS | CIN | N | P | N |  |
|  | | Normal |  |  |  |  |  |  |  | N | N | N |  |
| **128** | | Tumor | Male | 81 | Distal | Unknown | N | MSS | CIN | N | P | N |  |
|  | | Normal |  |  |  |  |  |  |  | N | P | N |  |
| **129** | | Tumor | Male | 58 | Distal | II | N | MSS | CIN | N | N | N |  |
|  | | Normal |  |  |  |  |  |  |  | N | N | N |  |
| **130** | | Tumor | Female | 80 | Distal | Unknown | N | MSS | CIN | N | P | N |  |
|  | | Normal |  |  |  |  |  |  |  | N | P | N |  |
| **131** | | Tumor | Female | 77 | Proximal | II | P | MSI | MSI | N | N | N |  |
|  | | Normal |  |  |  |  |  |  |  | N | N | N |  |
| **132** | | Tumor | Female | 72 | Distal | Unknown | N | MSS | CIN | N | P | P | 34,585.00 |
|  | | Normal |  |  |  |  |  |  |  | N | N | N |  |
| **133** | | Tumor | Female | 30 | Distal | Unknown | N | MSS | CIN | N | N | N |  |
|  | | Normal |  |  |  |  |  |  |  | N | N | N |  |
| **134** | | Tumor | Female | 81 | Distal | Unknown | N | MSS | CIN | N | P | N |  |
|  | | Normal |  |  |  |  |  |  |  | N | N | N |  |
| **135** | | Tumor | Female | 89 | Distal | II | N | MSS | CIN | N | N | N |  |
|  | | Normal |  |  |  |  |  |  |  | N | N | N |  |
| **136** | | Tumor | Male | 76 | Distal | Unknown | N | MSS | CIN | P | P | N |  |
|  | | Normal |  |  |  |  |  |  |  | N | P | N |  |
| **137** | | Tumor | Female | 60 | Distal | III | N | MSS | CIN | N | N | N |  |
|  | | Normal |  |  |  |  |  |  |  | N | N | N |  |
| **138** | | Tumor | Female | 35 | Distal | II | N | MSS | CIN | N | N | N |  |
|  | | Normal |  |  |  |  |  |  |  | N | N | N |  |
| **139** | | Tumor | Male | 57 | Distal | III | N | MSS | CIN | N | N | N |  |
|  | | Normal |  |  |  |  |  |  |  | N | N | N |  |
| **140** | | Tumor | Female | 51 | Proximal | II | P | MSI | MSI | N | P | N |  |
|  | | Normal |  |  |  |  |  |  |  | N | N | N |  |
| **141** | | Tumor | Male | 76 | Distal | Unknown | N | MSI | MSI | N | P | N |  |
|  | | Normal |  |  |  |  |  |  |  | N | N | N |  |
| **142** | | Tumor | Male | 61 | Distal | III | N | MSS | CIN | N | P | N |  |
|  | | Normal |  |  |  |  |  |  |  | N | N | N |  |
| **143** | | Tumor | Male | 72 | Distal | Unknown | N | MSS | CIN | N | P | N |  |
|  | | Normal |  |  |  |  |  |  |  | N | N | N |  |
| **144** | | Tumor | Female | 77 | Distal | III | N | MSS | CIN | N | P | N |  |
|  | | Normal |  |  |  |  |  |  |  | N | P | N |  |
| **145** | | Tumor | Male | 63 | Distal | II | N | MSS | CIN | N | P | P | 5,102.00 |
|  | | Normal |  |  |  |  |  |  |  | N | N | N |  |
| **146** | | Tumor | Female | 83 | Distal | II | N | MSS | CIN | N | P | N |  |
|  | | Normal |  |  |  |  |  |  |  | N | N | N |  |
| **147** | | Tumor | Male | 54 | Proximal | II | P | MSI | MSI | N | P | N |  |
|  | | Normal |  |  |  |  |  |  |  | N | N | N |  |
| **148** | | Tumor | Male | 74 | Distal | III | N | MSS | CIN | N | P | N |  |
|  | | Normal |  |  |  |  |  |  |  | N | N | N |  |
| **149** | | Tumor | Male | 64 | Distal | Unknown | N | MSS | CIN | N | P | N |  |
|  | | Normal |  |  |  |  |  |  |  | N | N | N |  |
| **150** | | Tumor | Female | 74 | Distal | Unknown | N | MSS | CIN | N | P | N |  |
|  | | Normal |  |  |  |  |  |  |  | N | N | N |  |
| **151** | | Tumor | Male | 67 | Distal | Unknown | N | MSS | CIN | N | P | N |  |
|  | | Normal |  |  |  |  |  |  |  | N | P | N |  |
| **152** | | Tumor | Male | 67 | Distal | II | N | MSS | CIN | N | P | N |  |
|  | | Normal |  |  |  |  |  |  |  | N | N | N |  |
| **153** | | Tumor | Female | 75 | Distal | Unknown | N | MSS | CIN | N | P | N |  |
|  | | Normal |  |  |  |  |  |  |  | N | N | N |  |
| **154** | | Tumor | Female | 55 | Unknown | III | N | MSS | CIN | N | P | N |  |
|  | | Normal |  |  |  |  |  |  |  | N | N | N |  |
| **155** | | Tumor | Male | 81 | Unknown | Unknown | N | MSS | CIN | N | P | N |  |
|  | | Normal |  |  |  |  |  |  |  | N | N | N |  |
| **156** | | Tumor | Female | 80 | Distal | III | N | MSS | CIN | N | P | N |  |
|  | | Normal |  |  |  |  |  |  |  | N | N | N |  |
| **157** | | Tumor | Female | 74 | Proximal | Unknown | P | MSI | MSI | P | P | N |  |
|  | | Normal |  |  |  |  |  |  |  | N | N | N |  |
| **158** | Tumor | Female | 79 | Unknown | III | P | MSS | CIMP | P | P | N |  |  |
|  | Normal |  |  |  |  |  |  |  | N | N | N |  |  |
| **159** | Tumor | Male | 55 | Unknown | Unknown | N | MSS | CIN | N | N | N |  |  |
|  | Normal |  |  |  |  |  |  |  | N | N | N |  |  |
| **160** | Tumor | Female | 66 | Distal | Unknown | N | MSS | CIN | N | N | N |  |  |
|  | Normal |  |  |  |  |  |  |  | N | N | N |  |  |
| **161** | Tumor | Male | 62 | Distal | Unknown | Unknown | MSS | na | na | na | N |  |  |
|  | Normal |  |  |  |  |  |  |  | N | N | N |  |  |
| **162** | Tumor | Female | 59 | Distal | Unknown | N | MSI | MSI | N | P | N |  |  |
|  | Normal |  |  |  |  |  |  |  | N | P | N |  |  |
| **163** | Tumor | Male | 77 | Unknown | Unknown | N | MSS | CIN | N | P | N |  |  |
|  | Normal |  |  |  |  |  |  |  | N | N | N |  |  |
| **164** | Tumor | Female | 50 | Unknown | Unknown | N | MSS | CIN | N | N | N |  |  |
|  | Normal |  |  |  |  |  |  |  | N | N | N |  |  |
| **165** | Tumor | Male | 64 | Unknown | Unknown | N | MSS | CIN | N | P | N |  |  |
|  | Normal |  |  |  |  |  |  |  | N | N | N |  |  |
| **166** | Tumor | Female | 74 | Unknown | Unknown | N | MSS | CIN | N | N | N |  |  |
|  | Normal |  |  |  |  |  |  |  | N | N | N |  |  |
| **167** | Tumor | Female | 71 | Unknown | Unknown | N | MSS | CIN | N | N | N |  |  |
|  | Normal |  |  |  |  |  |  |  | N | N | N |  |  |
| **168** | Tumor | Female | 84 | Distal | Unknown | N | MSS | CIN | P | P | N |  |  |
|  | Normal |  |  |  |  |  |  |  | N | N | N |  |  |
| **169** | Tumor | Male | 61 | Unknown | Unknown | P | MSS | CIMP | N | P | N |  |  |
|  | Normal |  |  |  |  |  |  |  | N | N | N |  |  |
| **170** | Tumor | Female | 68 | Unknown | Unknown | N | MSS | CIN | N | P | N |  |  |
|  | Normal |  |  |  |  |  |  |  | N | N | N |  |  |
| **171** | Tumor | Female | 77 | Unknown | Unknown | P | MSS | CIMP | N | P | N |  |  |
|  | Normal |  |  |  |  |  |  |  | N | P | N |  |  |
| **172** | Tumor | Female | 56 | Unknown | Unknown | N | MSS | CIN | P | N | N |  |  |
|  | Normal |  |  |  |  |  |  |  | N | N | N |  |  |
| **173** | Tumor | Female | 57 | Distal | Unknown | P | MSS | CIMP | P | N | N |  |  |
|  | Normal |  |  |  |  |  |  |  | N | N | N |  |  |
| **174** | Tumor | Female | 58 | Distal | Unknown | N | MSS | CIN | N | P | N |  |  |
|  | Normal |  |  |  |  |  |  |  | N | N | N |  |  |
| **175** | Tumor | Male | 55 | Distal | Unknown | N | MSS | CIN | P | N | N |  |  |
|  | Normal |  |  |  |  |  |  |  | N | N | N |  |  |
| **176** | Tumor | Male | 61 | Distal | Unknown | N | MSS | CIN | N | P | N |  |  |
|  | Normal |  |  |  |  |  |  |  | N | P | N |  |  |
| **177** | Tumor | Male | 49 | Distal | Unknown | N | MSS | CIN | N | N | N |  |  |
|  | Normal |  |  |  |  |  |  |  | N | N | N |  |  |
| **178** | Tumor | Female | 88 | Unknown | Unknown | N | MSS | CIN | N | P | N |  |  |
|  | Normal |  |  |  |  |  |  |  | N | P | N |  |  |
| **179** | Tumor | Female | 81 | Unknown | Unknown | P | MSS | CIMP | P | N | N |  |  |
|  | Normal |  |  |  |  |  |  |  | N | N | N |  |  |
| **180** | Tumor | Female | 76 | Unknown | Unknown | N | MSS | CIN | N | P | N |  |  |
|  | Normal |  |  |  |  |  |  |  | N | N | N |  |  |
| **181** | Tumor | Female | 68 | Distal | Unknown | N | MSS | CIN | P | P | N |  |  |
|  | Normal |  |  |  |  |  |  |  | N | P | N |  |  |
| **182** | Tumor | Male | 62 | Unknown | Unknown | N | MSS | CIN | N | P | N |  |  |
|  | Normal |  |  |  |  |  |  |  | N | P | N |  |  |
| **183** | Tumor | Female | 91 | Unknown | Unknown | N | MSS | CIN | P | P | N |  |  |
|  | Normal |  |  |  |  |  |  |  | N | N | N |  |  |
| **184** | Tumor | Male | 81 | Distal | Unknown | P | MSS | CIMP | N | P | N |  |  |
|  | Normal |  |  |  |  |  |  |  | N | N | N |  |  |
| **185** | Tumor | Female | 58 | Unknown | Unknown | N | MSS | CIN | P | N | N |  |  |
|  | Normal |  |  |  |  |  |  |  | N | N | N |  |  |
| **186** | Tumor | Male | 87 | Unknown | Unknown | N | MSS | CIN | N | N | N |  |  |
|  | Normal |  |  |  |  |  |  |  | N | N | N |  |  |
| **187** | Tumor | Female | 59 | Distal | Unknown | N | MSS | CIN | P | N | N |  |  |
|  | Normal |  |  |  |  |  |  |  | N | N | N |  |  |
| **188** | Tumor | Male | 86 | Unknown | Unknown | P | MSI | MSI | N | P | N |  |  |
|  | Normal |  |  |  |  |  |  |  | N | N | N |  |  |
| **189** | Tumor | Female | 71 | Unknown | Unknown | N | MSS | CIN | N | N | N |  |  |
|  | Normal |  |  |  |  |  |  |  | N | N | N |  |  |
| **190** | Tumor | Male | 84 | Unknown | Unknown | N | MSS | CIN | N | P | N |  |  |
|  | Normal |  |  |  |  |  |  |  | N | N | N |  |  |

CIMP*: CpG island methylator phenotype (CIMP); MSI: Microsatellite instability; MSS: Microsatellite stability; CIN: Chromosomal instability; CMV: Cytomegalovirus; EBV: Epstein-Barr virus; SG: *Streptococcus gallolyticus*; “P” indicates positive and “N” indicates negative.
